# Comparative analysis of miRNAs and their targets across four plant species

**DOI:** 10.1186/1756-0500-4-483

**Published:** 2011-11-08

**Authors:** Dorina Lenz, Patrick May, Dirk Walther

**Affiliations:** 1 Max Planck Institute for Molecular Plant Physiology, Am Mühlenberg 1, 14476 Potsdam-Golm, Germany; 2 Luxembourg Centre for Systems Biomedicine University of Luxembourg, Luxembourg; 3 LGC Genomics GmbH, Ostendstrasse 25, 12459 Berlin, Germany; 4 Institute for Systems Biology, Seattle, WA, USA

**Keywords:** plants, miRNA, miRNA targets, conservation, next generation sequencing

## Abstract

**Background:**

MicroRNA (miRNA) mediated regulation of gene expression has been recognized as a major posttranscriptional regulatory mechanism also in plants. We performed a comparative analysis of miRNAs and their respective gene targets across four plant species: *Arabidopsis thaliana (Ath), Medicago truncatula(Mtr), Brassica napus (Bna)*, and *Chlamydomonas reinhardtii (Cre)*.

**Results:**

miRNAs were obtained from mirBase with 218 miRNAs for Ath, 375 for Mtr, 46 for Bna, and 73 for Cre, annotated for each species respectively. miRNA targets were obtained from available database annotations, bioinformatic predictions using RNAhybrid as well as predicted from an analysis of mRNA degradation products (degradome sequencing) aimed at identifying miRNA cleavage products. On average, and considering both experimental and bioinformatic predictions together, every miRNA was associated with about 46 unique gene transcripts with considerably variation across species. We observed a positive and linear correlation between the number miRNAs and the total number of transcripts across different plant species suggesting that the repertoire of miRNAs correlates with the size of the transcriptome of an organism. Conserved miRNA-target pairs were found to be associated with developmental processes and transcriptional regulation, while species-specific (in particular, Ath) pairs are involved in signal transduction and response to stress processes. Conserved miRNAs have more targets and higher expression values than non-conserved miRNAs. We found evidence for a conservation of not only the sequence of miRNAs, but their expression levels as well.

**Conclusions:**

Our results support the notion of a high birth and death rate of miRNAs and that miRNAs serve many species specific functions, while conserved miRNA are related mainly to developmental processes and transcriptional regulation with conservation operating at both the sequence and expression level.

## Background

The discovery of microRNAs (miRNAs) in different kingdoms and in many species prompted comparative analyses to identify those miRNAs that are more strongly conserved than others and to understand whether their main functional role is associated with species-specific or universally occurring processes or both. Animal miRNAs have been reported to be involved in developmental timing, cell death, cell proliferation, haematopoiesis, and patterning of the nervous system [1], i.e. primarily developmental processes. MicroRNAs involved in these processes were also found to be conserved across species [2]. Genes involved in basic cellular maintenance functions are less often miRNA targets [3]. Many miRNA families have also been conserved across different plant lineages including mosses, gymnosperms, moncotes, and dicots [4-6]. However, with modern sequencing technologies that allow miRNAs do be identified at increased breadths, it was also noted that the number of species-specific miRNAs is greater than the number of conserved miRNAs [7,8]. Thus, a high birth and death rate of miRNAs has been postulated [7]. The highly dynamic nature of miRNA evolution was also confirmed recently in a comparative analysis of the closely related Arabidopsis species *A.thaliana *and *A.lyrata *[9]. A substantial number of miRNAs was found to be species-specific, despite the only recent separation of the two species. No event of miRNA conservation between plants and animals has yet been found [10]. As also the miRNA processing and targeting differs substantially, it has been concluded that the miRNA mechanism has evolved separately in animals and plants from common ancestral siRNA machinery [5].

In this study, we carried out a comparative analysis of miRNAs and their targets across the four plant species *Arabidopsis thaliana *(Ath), *Brassica napus*(Bna) - both members of the brassicaceae family, *Medicago truncatula *(Mtr) - a legume, and *Chlamydomonas reinhardtii *(Cre) - a single cell alga. The choice of plant species was motivated by several research projects conducted at the Max Planck Institute for Molecular Plant Physiology. The unifying goal of these studies was to identify functional miRNAs, to profile known miRNAs with regard to their abundance and to potentially discover novel miRNAs by applying the Solexa/Illumina Next Generation Sequencing (NGS) technology to RNA extractions for the different plant systems exposed to different conditions. More specifics and results regarding these studies can be found in [11] and [12], and in the Method section. Recently, so-called degradome sequencing was established as a powerful experimental approach to detect miRNA targets [13] and corresponding bioinformatic data processing pipelines introduced [14]. Here, the cleavage products generated upon miRNA induced mRNA target cleavage are specifically identified thereby allowing those miRNA-target pairs to be identified for which cleavage is the mode of action while not detecting those targets that are under translational repression. Degradome data have also been used in the current study.

## Methods

### miRNA, cDNA, EST sequence data

For the four investigated plant species, we obtained mature miRNA sequences and stem-loop sequences associated with miRNA precursors from miRBase release 15 http://www.mirbase.org[15] yielding 218 miRNAs for Ath, 375 for Mtr, 46 for Bna, and 73 for Cre, respectively. No miRNA-star sequences were considered for analysis. For Ath, cDNA sequence information was obtained from The Arabidopsis Information Resource (TAIR, http://www.arabidopsis.org), genome release 9 [16]. For Bna, assembled contigs were retrieved from PlantGDB http://www.plantgdb.org/. For Mtr, sequence and annotation information was obtained from The Medicago Genome Sequence Consortium (MGSC, http://www.medicago.org), and here referred to as "Mt3.0". Sequences for Cre were downloaded from the DOE Joint Genome Institute using genome assembly v4.0 and Augustus v5.0 gene models http://genome.jgi-psf.org.

### Conditions for experimental data; small RNA, degradome sequencing data

We used smallRNA sequencing data obtained and published for Ath under eight [11,17] and for Mtr under two different experimental conditions [12]. The specific conditions were for Mtr: a) treatment with the symbiotic fungus mycorrhiza ("Myc") and b) treatment without the fungus ("N-Myc"). The eight conditions for Ath were: full nutrition ("FN"), phosphate starvation ("P"), phosphate starvation after three hours phosphate re-addition ("P+3 h"), nitrogen starvation ("N"), nitrogen starvation after three hours nitrogen re-addition ("N+3 h") (all from [11]), and FN from root cells ("root+p"), phosphate starvation from root cells ("root-p"), and phosphate starvation from shoot cells ("shoot-p") (from [17]). In total, 15.8 Mill small RNA reads were sequenced for Ath and 13.6 Mill reads for Mtr (2 conditions ("Myc", "N-Myc"). Degradome data to experimentally identify miRNA targets by detecting miRNA induced cleavage products from four conditions in Ath ("FN", "P-12 h", "P-48 h" and "N-48 h") and two conditions in Mtr ("Myc", "N-Myc") were used. For experimental details see [11,12].

### Normalization of expression data

Normalization of expression values per condition was done to adjust for variable sequencing depth between samples. The sequencing reads mapping on annotated miRNA were normalized to reads per million (RPM) per experimental condition: number of reads per gene/number of total reads * 1E6.

### Criteria for conserved miRNAs

For analyzing the conservation of miRNA families across species, we performed a pairwise global sequence alignment of all single mature miRNA sequences with the program Align0 [18]. Sequence pairs were considered conserved if the sequence identity was greater than 75, if there was a perfect match of seed sequence (6 nt, positions 2-7), and the two respective identifiers of the pair were classified by miRBase to be in the same MIRNA family.

### miRNA-target relationships

Verified miRNA-target relationships were extracted from several sources: Supplementary Data of [19] (500 targets), 530 targets in total from the Arabidopsis Small RNA Project, "ASRP" (http://asrp.cgrb.oregonstate.edu, [20]), experimental data reported in Supplementary Table two and three of [13], referred to here as "degradomeG" (60 targets), and degradome sequencing data for Ath and Mtr from in-house experiments, called "degradome" (1,154 targets) [11,12]. To identify miRNA-target relationships from degradome data, the CleaveLand algorithm was used [14,21]. miRNA-targets were further predicted using the program RNAhybrid [22]. The mature miRNA sequence data from miRBase and, on the potential target side, the downloaded cDNAs or assembled ESTs mentioned above were used as input. We used the parameter settings described in [19]. We required the minimum free energy of hybridization to be greater than 70% compared to perfect match hybridization; i.e. in concordance with the initial threshold used in [19]. Note that for the final set, the authors in [19] used a stricter 75% mfe cutoff. In Arabidopsis and using a 75% mfe threshold level, we obtained 2,967 unique targets transcripts for 218 miRNAs. All RNAhybrid predictions with additional score and mfe information for all four plant species are provided in tabular format as supplementary material (Additional File 1).

### GO annotations

Gene Ontology (GO) annotation files were downloaded: for Ath from TAIR [16], for Cre from the DOE Joint Genome Institute http://genome.jgi-psf.org/Chlre4/Chlre4.download.ftp.html, and for Mtr from http://www.medicago.org/genome/downloads/Mt3/. Annotations for Bna were assigned by copying the GO slim term from TAIR for the best hit from a BLAST run against Ath. The calculation of over-representation of GO terms was done by applying the Fisher's Exact Test for count data and the *p*-values for Molecular Functions and Biological Processes were adjusted for multiple testing applying the Benjamini-Hochberg method [23].

## Results

### Overview of the miRNA and target statistics

First, we provide an overview of the statistics of miRNAs, their targets, and the genomic context in the respective plant species (Table 1). We based our analyses on the miRNAs deposited in miRBase (v15, see Methods). Interestingly, for the three well annotated species (Ath, Mtr, Cre), a positive correlation was found between the number of miRNAs and the genome size (Pearson correlation coefficient *r *= 0.91), and even more strongly to the number of transcripts encoded in those genomes (*r *= 0.99), albeit with only three species, significance cannot yet be established. Thus, the repertoire of miRNAs appears to scale with the size of the transcriptome that is to be regulated. Since for Bna only assembled EST reads were available that are likely overestimating the number of transcripts, Bna was not included in this statistic. However, the average number of targets per miRNA appears to differ quite substantially between species (Table 1). Across all species, every miRNA targets, on average, 46 transcripts indicating a rather large target set per miRNA. In Ath, about one fifth of all transcripts is predicted to be targeted by the known miRNAs, whereas in Cre, about half of all transcripts are under miRNA control, while the percentage is about 10% in Mtr, and even less for Bna (0.7%), although it needs to be noted again that the number of transcripts may be inflated in Bna. Furthermore, miRNAs in Bna were found primarily via sequence similarity searches. Thus, the Bna-specific miRNA set may be missing altogether. Target assignments came primarily from RNAHybrid predictions (94%), and the remaining targets from public domain resources and the used degradome-sequencing data (see Methods). Of the 885 miRNA-target relationships in Ath (269 in Mtr) detected in the degradome dataset by the Cleaveland program (see Methods), 274 were also predicted by RNAHybrid (176 in Mtr) (Note: comparison was done independently of the actual cleavage position). Thus, many real, experimentally determined targets were actually missed by RNAHybrid, especially in Ath.

**Table 1:** Statistics overview.

	Ath	Bna	Cre	Mtr
**Genome size (Mbp)**	157^a^	566^a^	121^b^	500^c^
**cDNAs/contigs**	33,088	131,259	16,888	53,423
**miRNAs**	218	46	73	375
**miRNA:target pairs**	9,462	1,358	15,531	12,183
**distinct transcript** **targets**	6,788	1,050	8,608	5,304
**average targets per** **miRNA**	31.1	22.1	117.9	15.5

Statistics of genomic properties and miRNA, miRNA-target counts in the four plant species used in this study.

Data taken from a - [25], b - [26], c - [27]

As reported for plant miRNA target action earlier [24], most miRNA target sites were found to fall within the coding regions (86% in Ath), whereas the 5' and 3'UTR regions are targeted by approximately 7% (in Ath).

### Conservation of miRNAs and their targets

**Figure 1:**
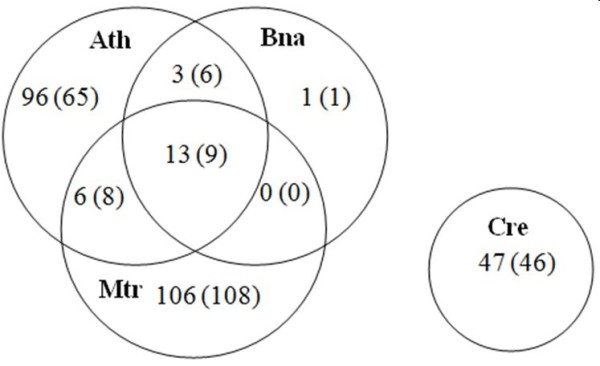
**Venn diagram of miRNA families conserved between species**. Numbers in brackets refer to conserved miRNA-target relationships.

Relative to Ath, Bna is evolutionarily the closest species with their supposed common ancestor dating back ~40 Mya, followed by Mtr (~110 Mya), and Cre as the evolutionarily farthest species relative to Ath (~475 Mya) (data taken from [9]). According to miRBase (v15), the 712 individual miRNAs belong to 272 miRNA families. For the 47 miRNA families present in Cre, no event of conservation was found with a miRNA family from any of the three other organisms, and there was no conservation reported with any other plant species yet either [5,9]. Only a very small fraction of miRNA families is present in more than one organism, 22 miRNA families in total. A larger fraction of miRNAs appears to be species specific (Figure 1). Bna is an exception, for which we found only one species specific miRNA family, but this may be due to the incomplete genome and miRNA search strategies based on sequence homology to other plant species.

### miRNA-target relationships in Arabidopsis thaliana

**Figure 2:**
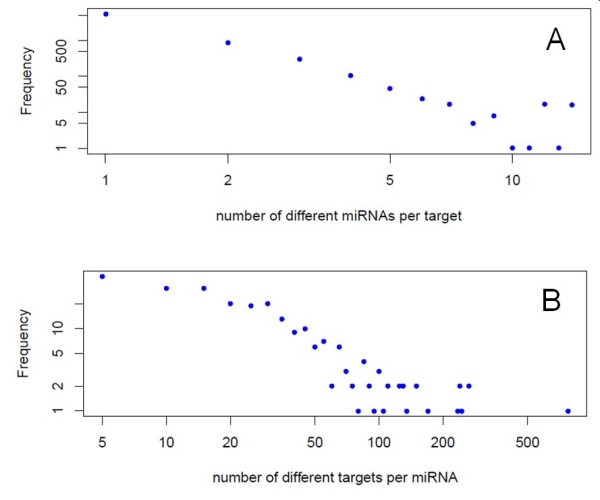
**Frequency distribution of miRNA-target relationships in Ath**. Double-logarithmic plot of A) number of different miRNAs per target transcript, B) number of different target transcripts per miRNA and their respective frequency of occurrence.

In Ath, most of the 6,788 target transcripts associated with 5,690 genes are targeted by only one miRNA with the frequency of multiple hits decreasing rapidly with increasing number of miRNA targeting a mRNA, observed to follow a power-law (linear in double-logarithmic diagram) (Figure 2A). Transcripts targeted by 3 or more miRNAs were observed to be preferentially associated with developmental processes (GO-slim enrichment *p*-value (FDR multiple testing corrected) of 1.86E-6), response to abiotic and biotic stimulus (*p *= 3.46E-4), transcription (*p *= 4.15.E-3), and response to stress (*p *= 1.03E-3), compared to those transcriptions targeted by only a single miRNA. Conversely, for the number of targets hit by a single miRNA a relative plateau is observed up until about 20 different targets per miRNA decreasing also in a power-low fashion beyond this number.

### Properties of conserved versus non-conserved miRNA

Conserved miRNA families were found to target on average more gene transcripts - with the average number of targets summed up across the three species Ath, Bna, and Mtr amounting to 161.4 - than their non-conserved counterparts (106.2), *p *= 0.073 (Mann-Whitney test). Based on the available quantitative data of miRNA expression via normalized read counts (see Methods), conserved miRNAs were found to be expressed at higher levels than non-conserved miRNAs. In Ath, the average log-2 expression value for conserved miRNAs was 9.25 and significantly higher than the corresponding value for non-conserved miRNAs (3.92, *p *= 2.2e-5), observed similarly in Mtr with 7.39 for conserved vs. 4.94 average log-2 expression level for non-conserved miRNAs, albeit significance could not be established (*p *= 0.21).

Regarding biological processes particularly associated with genes targeted by conserved miRNAs, developmental and transcription-related processes are overrepresented (Table 2). Species-specific; i.e. non-conserved miRNAs, are predominantly associated with signal transduction and response to stress processes, as were "unknown biological processes", in fact this category was the most significant one (Table 2).

**Table 2:** Biological process involvement of conserved/non-conserved miRNAs.

Conserved targets		Species specific targets	
**p-value**	**GO-Process Term**	**p-value**	**GO Process Term**

2.31E-29	Developmental processes	4.91E-42	Unknown biological processes
9.31E-05	Transcription	2.68E-03	Signal transduction
2.68E-03	Other metabolic processes	3.90E-03	Response to stress
1.31E-02	Other cellular processes	3.23E-02	Transport
2.45E-02	Electron transport or energy pathway	5.73E-02	Protein metabolism

GO-slim Process terms enrichment analysis using the Fisher's exact test and applied multiple testing correction (see Methods).

### Conservation of miRNA expression levels

**Figure 3:**
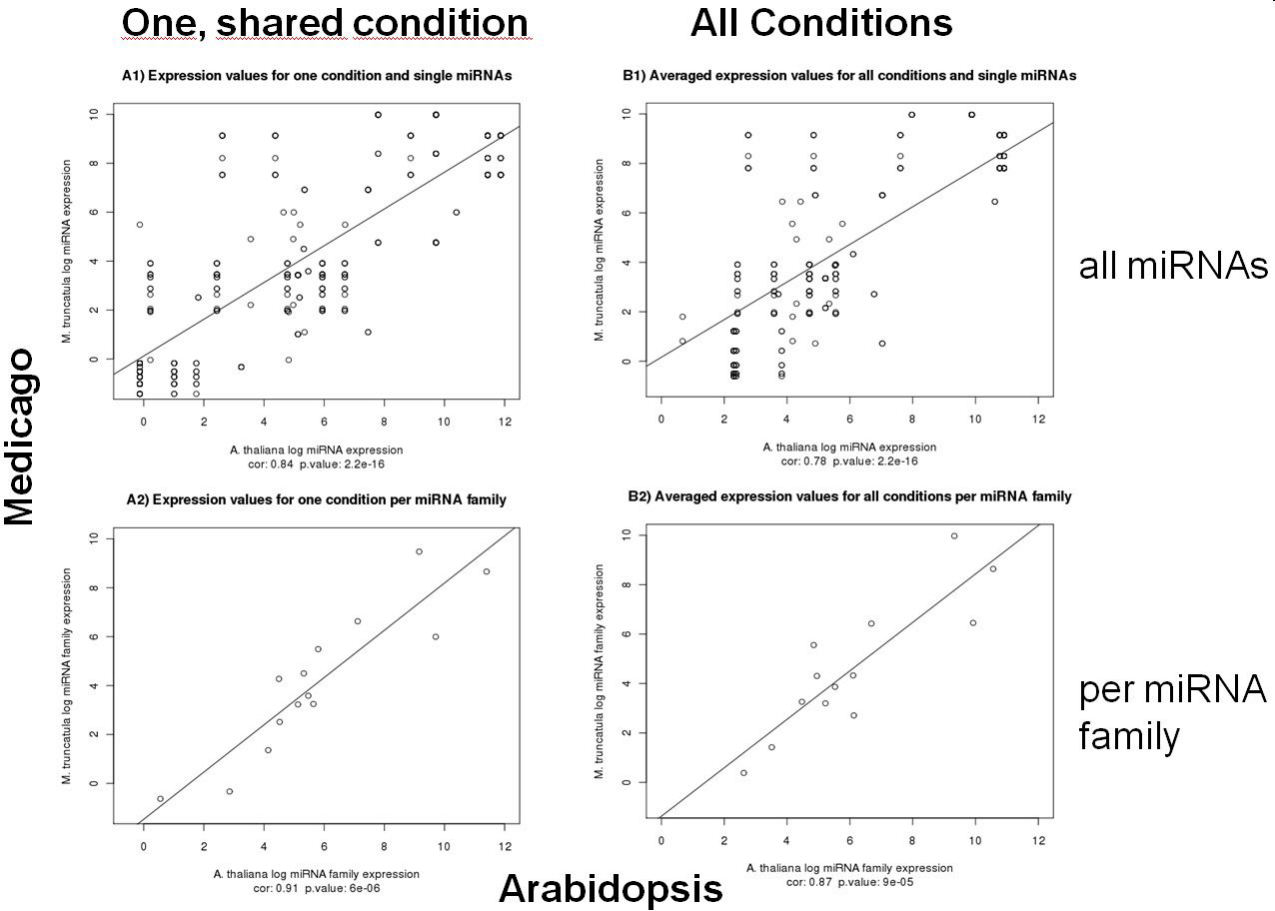
**Comparison of miRNA expression levels in Ath and Mtr based on normalized read counts from Solexa/Illumina sequencing data**. Graphs A1 and A2 show the results for the one experimental condition that was directly comparable between Ath and Mtr (Ath: phosphate-starvation, Mtr - non-mycorrhiza state). Graphs B1 and B2 show the averaged expression levels across all conditions available for the respective species. The upper row (A1, B1) display the results for all individual miRNAs, the lower row (A2, B2) shows expression levels averaged over all miRNAs belonging to the same miRNA family. Values refer to log-2 transformed normalized read counts.

The available quantitative miRNA expression data derived from normalized read counts allowed us to compare expression levels of conserved miRNAs in the species Ath and Mtr and to probe whether expression levels are also conserved for sequence-conserved miRNAs. Among the various experimental conditions applied in the studies involving the two species, one was nearly equivalent between them: the phosphate-starvation condition in Ath and the non-mycorrhiza conditions in Mtr. Mycorrhiza facilitates phosphate uptake from the soil, thus non-mycorrhiza plants are exposed to phosphate starvation conditions. For both individual miRNAs and averaged per miRNA family, a strong and significant positive correlation was found (Figure 3). Even when comparing average expression levels across all available 8 experimental conditions in Ath and two in Mtr, relative expression levels of sequence-conserved miRNAs appear to be preserved in the two different species as well. It appears plausible that highly expressed miRNAs target more transcripts than miRNAs expressed at low levels. Indeed, a positive correlation between the number of targets and expression level was observed in Ath (*r *= 0.29, *p *= 0.02), while no correlation was evident in Mtr (*r *= - 0.02, *p *= 0.95). Thus, the true nature of this relationship still remains to be established.

## Discussion

We performed a comparative analysis of miRNAs in four different plant species (Ath, Bna, Mtr, and Cre). Our results confirm previous results that miRNA evolution appears to be rapid suggesting a significant participation of miRNAs in species-specific processes [8,9]. The observation that species-specific miRNAs and their targets appear to be involved in processes involving interactions with the environment, such as signal transduction and stress response (Table 2) supports the notion that miRNAs are an important level of regulation at the speciation level as every species will have their very own environment to cope with. The observation that genes involved in "unknown biological processes" were also found overrepresented in the set of target genes of non-conserved miRNAs may either suggest that there are still many species-specific genes not properly characterized yet, or that those miRNA-target associations are spurious in the sense that the annotation of the genes and/or the identification of the miRNA may have been incorrect.

Small RNA sequencing data was analyzed to assess conservation not only at the sequence, but also at the expression level with the conclusion that miRNA expression is conserved as well. Therefore, it may be worthwhile to compare the respective cis-regulatory regions associated miRNA genes across different species and to investigate evolutionary differences and conservation patterns.

Further improvements also seem possible on the bioinformatic target prediction side. While it is clear that *in silico *methods may yield more predictions than miRNA-target pairs detected experimentally - as they depend on the miRNA actually being expressed - ideally, all of the experimentally found miRNA-target pairs would also be found by *in silico *methods.

## Conclusion

Gene expression regulation via miRNAs in plants appears to scale with genome size and to play a predominant role in species specific adaptation processes. In cases of miRNA conservation not only is the sequence conserved, but also their expression with targeted processes associated with general, developmental programs.

## Abbreviations

Ath: Arabidopsis thaliana; Bna: Brassica napus; Mtr: Medicago truncatula; Cre: Chlamydomonas reinhardtii

## Competing interests

The authors declare that they have no competing interests.

## Authors' contributions

DW conceived and supervised the study. DL performed all computations and analyses. PM co-supervised the study, contributed the data processing and analysis. All authors wrote the manuscript. All authors read and approved the final version of the manuscript.

## Supplementary Material

Additional file 1 **Contents: RNAhybrid-based miRNA-target pairs for all four plant species and additional score, minimum-free-energy, mfe-ratio (actual mfe divided by mfe for perfect match) and sequence detail information**. Note that the degradome-targets are available from their respective original publications [11,12]. Click here for file
